# Physiotherapy management of patients with trunk trauma: A state-of-the-art review

**DOI:** 10.4102/sajp.v76i1.1406

**Published:** 2020-06-11

**Authors:** Helena van Aswegen

**Affiliations:** 1 Department of Physiotherapy, Faculty of Health Sciences, University of the Witwatersrand, Johannesburg, South Africa

**Keywords:** chest trauma, abdominal trauma, physiotherapy, quality of life, rehabilitation, outcome measures

## Abstract

**Background:**

Trauma injury remains a significant health risk for all on a global level. Patients with trunk trauma suffer blood loss, inflammation and hypoxia and are at risk of developing respiratory and musculoskeletal complications during their recovery. Physiotherapists are an integral part of the interprofessional team that manages patients who sustain trunk trauma.

**Objectives:**

To describe the physiotherapy management of adult patients with trunk trauma, their quality of life, post-discharge rehabilitation service provision, and outcome measures used in the physiotherapy management.

**Method:**

A non-systematic narrative review of published literature was performed.

**Results:**

Mobilisation, functional exercises, deep breathing exercises and active coughing are used to optimise patients’ respiratory and musculoskeletal functioning. Some physiotherapists educate patients on the use of pain management strategies to reduce discomfort from rib fractures, surgical sites and intercostal drainage bottle tubing. Survivors of trunk trauma experience limitations in physical function up to two years. Little is known about post-discharge rehabilitation service provision to these patients after discharge. Few physiotherapists use outcome measures as part of their daily clinical practice.

**Conclusion:**

Physiotherapy management of patients with blunt or penetrating trunk trauma during hospitalisation and after discharge is a field of clinical practice that is rich for high-quality research related to service provision, cost analysis and interventions used.

**Clinical implications:**

Physiotherapy clinicians and researchers can use the findings of this review as a guide to their management of adult patients recovering from trunk trauma.

## Introduction

Globally, more people suffer death and disability as a result of traumatic injury that poses a significant health risk to all (World Health Organization 2018). The 2014 WHO Global Status Report on Violence Prevention stated that road traffic accidents injure approximately 50 million people worldwide annually and will be the seventh leading cause of death by 2030. According to the same report, interpersonal violence is the fourth leading cause of death for people aged 15–44 years (World Health Organization 2014). Thoracic injuries contribute up to 75% of trauma-related deaths (World Health Organization 2014).

In South Africa (SA), traumatic injury was labelled a malignant epidemic in 1991 and is still being referred to as such (John & Mathsoba 2015). Approximately 48 000 South Africans are killed annually because of traumatic injury and a further 3.5 million seek healthcare as a result of traumatic injury on a yearly basis (John & Mathsoba 2015). This high rate of traumatic injury in SA can be ascribed to rising levels of poverty and unemployment, abuse of drugs and alcohol, easy access to weapons and exposure to violence in the community, and a weak culture of law enforcement (John & Mathsoba 2015). In 2012, 54 870 firearm-related injuries occurred in SA and individual patient costs because of firearm injury alone averaged R24 945 (Martin et al. 2017).

The trunk is described as the body without the head and limbs; therefore, the trunk consists of the abdomen, back and thorax (Taber’s Cyclopedic Medical Dictionary 2013). Injury to the trunk results from blunt or penetrating trauma (Van Aswegen, Morrow & Van Aswegen 2015). Blunt trauma may result in injury to the chest wall, lung parenchyma and pleura, tracheobronchial tree, heart and great vessels of the chest, oesophagus and the diaphragm and/or burst or crush injury to the abdominal organs, but the skin on the chest and abdominal walls stays intact (Van Aswegen, Morrow & Van Aswegen 2015). Conversely, penetrating trauma results in penetration of an object through the skin and soft tissue of the thoracic wall into the pleural space and subsequent loss of negative intrapleural pressure from the thoracic cavity. Penetrating thoracic injury can be the most lethal of injuries. Penetrating abdominal injuries are not immediately life-threatening unless a major blood vessel is damaged (Van Aswegen, Morrow & Van Aswegen 2015). A report from a trauma centre in KwaZulu-Natal province showed that causes of death following thoracic trauma were almost equally spread between blunt and penetrating trauma (55.7% and 44.3%, respectively) (Moodley et al. 2014).

Major thoracic trauma is defined as the presence of an open pneumothorax, tension pneumothorax, massive haemothorax, flail chest injury and/or pulmonary contusion (Ludwig & Koryllos 2017). Other injuries associated with thoracic trauma include spinal cord injury, splenic and hepatic injury in the presence of rib fractures, brachial plexus injury (associated with fractures of ribs 1–3), injury of the sub-clavian artery and the development of Horner’s syndrome (interruption of sympathetic nerve supply to the eye resulting in a constricted pupil, partial ptosis and hemi-facial sweating) (Bulger et al. 2000; Van Aswegen, Morrow & Van Aswegen 2015). Those at increased risk of developing complications following major thoracic trauma have three or more rib fractures, are 65 years or older, used anticoagulant therapy pre-injury, have previously diagnosed chronic respiratory disease or cardiovascular disease, and/or have arterial oxygen saturation of less than 90% on casualty department admission (Battle et al. 2013). The development of pneumonia and/or respiratory failure significantly increases the risk of mortality for those with major thoracic trauma (Battle, Hutchings & Evans 2012).

On casualty department admission, a patient with major trunk trauma is managed according to Advanced Trauma Life Support guidelines (Thim et al. 2012). In-hospital management in an intensive care unit (ICU), high care unit or ward setting includes pharmacological pain management, placement of intercostal drain/s (ICD), supportive therapy in the form of intubation and mechanical ventilation or management through non-invasive ventilation (NIV) and oxygen therapy, physiotherapy, damage control surgery to the abdomen, re-look laparotomy procedures and surgical rib fixation, if indicated (Van Aswegen, Morrow & Van Aswegen 2015; Van Aswegen et al. 2019). The body’s response to traumatic injury, blood loss and surgery is activation of a major acute inflammatory response because of overproduction and release of pro-inflammatory cytokines, reactive oxidant species and nitric oxide into the blood circulation and suppression of anti-inflammatory cell activity (Puthucheary et al. 2018). This accentuated pro-inflammatory status contributes to muscle protein breakdown and wasting (Puthucheary et al. 2018; Van Aswegen et al. 2010). Blood oxygen levels are low in patients who sustain traumatic injury because of reduced erythropoietin production and phlebotomy procedures in the ICU setting (Backman et al. 2018; Scharte & Fink 2003). Low oxygen levels further increase pro-inflammatory cell activity creating chronic low-grade systemic inflammation that leads to insulin resistance (Puthucheary et al. 2018). The systemic inflammatory response syndrome may lead to the development of sepsis and/or multiple organ dysfunction or failure (Puthucheary et al. 2018; Van Aswegen, Morrow & Van Aswegen 2015). Those with multiple organ dysfunction develop severe muscle wasting early and rapidly during their ICU stay (Connolly et al. 2016).

Physiotherapists play an important and integral role as members of the interprofessional team that manages patients with traumatic injury (Curtis et al. 2016; Kourouche et al. 2018; Unsworth et al. 2015). The implementation of clinical practice guidelines or protocolised pathways (including physiotherapy) in the management of patients with major thoracic and abdominal trauma is beneficial as it results in improved clinical outcomes such as reduced mortality and rates of pneumonia, and decreased ICU and hospital length of stay (LOS) (Curtis et al. 2016; Hanekom, Louw & Coetzee 2012; Todd et al. 2006; Unsworth et al. 2015).

## Objectives of review

Little is known about the types of physiotherapy interventions used or their effectiveness in the management of patients recovering from trunk trauma during their in-hospital stay and after discharge from hospital. The aims of this narrative review are to describe (1) the physiotherapy management of adult patients admitted to acute care facilities with trunk trauma, (2) the quality of life (QOL) of adult survivors of trunk trauma and rehabilitation service provision and (3) outcome measures used in the management of patients with trunk trauma.

## Methods

A non-systematic narrative review of the published literature was conducted (Green, Johnson & Adams 2006).

Search engines used included PubMed, Scopus, Google Scholar, EBSCOhost and PEDro. Condition-specific search terms used included ‘trunk trauma’, ‘thoracic trauma’, ‘chest trauma’, ‘rib fractures’, ‘pneumothorax’, ‘haemothorax’, ‘multiple rib fractures’, ‘flail chest’, ‘blunt trauma’, ‘penetrating trauma’, ‘abdominal trauma’ and ‘severity of injury’. Population-specific search terms were limited to ‘adults’. Intervention search terms used were ‘physiotherapy’, ‘physical therapy’, ‘respiratory function’, ‘mobilisation’, ‘exercise capacity’, ‘physical function’, ‘function’ and ‘QOL’. Databases were searched from the date of inception to December 2019 and articles published in the English language that described relevant components of physiotherapy management of patients with trunk trauma were considered for inclusion in this review.

### Ethical consideration

The author was not exposed to any confidential information from patients and the articles reviewed are available in the public domain. Therefore, it was not necessary to apply for permission from an ethics committee to conduct this review.

## Results

Fourteen articles were included in this review. The study designs of these articles ranged from randomised trials (*n* = 3), cohort studies (*n* = 3), longitudinal studies (*n* = 2), cross-sectional studies (*n* = 3), narrative reviews (*n* = 1) and expert opinion papers (*n* = 1) to qualitative reports (*n* = 1). The findings of this review are reported under the following subheadings below: physiotherapy interventions used to optimise respiratory function; physiotherapy interventions used to optimise musculoskeletal function; physiotherapy interventions used to manage pain; QOL of survivors of trunk trauma and rehabilitation service provision; and outcome measures.

### Physiotherapy interventions used to optimise respiratory system function

Body function and structure impairments (International Classification of Functioning, Disability and Health [ICF]) that patients with trunk trauma present with clinically are summarised in Table 1. These impairments pose limitations on patients’ abilities to participate in rehabilitation activities and activities of daily living which places them at increased risk of morbidity and mortality. The principles of physiotherapy management of such patients include adequate humidification of the airways; mobilisation and removal of excessive retained secretions from the tracheobronchial tree; enhancement of the patient’s cough effort; increasing and restoring lung capacities, volumes and compliance; improving oxygenation; and improving respiratory muscle strength to assist with weaning of patients from mechanical ventilation support (Van Aswegen, Morrow & Van Aswegen 2015).

**TABLE 1:** Body function and structure impairments commonly observed in patients with trunk trauma.

Impairments	Causes of impairments
Insufficient inspiratory lung capacity	Pain from rib fractures
	Postoperative pain following abdominal or thoracic surgery
	Retention of air and/or blood in the pleural cavity
Poor cough effort	Pain from rib fractures
	Postoperative pain following abdominal or thoracic surgery
Insufficient lung capacity, compliance and volumes	Abdominal distention and splinting of diaphragm movement
	Destabilisation of the chest wall
	Pulmonary contusion
	Retention of air and/or blood in the pleural cavity
Impaired gas exchange and oxygenation	Loss of lung volume
	Pulmonary contusion

The earliest account of physiotherapy management of patients with penetrating trunk trauma is the expert opinion paper by Hayse-Gregson (1973) from Chris Hani Baragwanath Hospital. She described the use of intervention strategies such as breathing exercises (diaphragmatic breathing, localised breathing on the affected side and bilateral lateral costal breathing); active coughing; a variety of active arm, shoulder and thoracic mobility exercises; and mobilisation away from the bedside for patient management. Physiotherapy was provided twice daily in the form of individual and group treatment sessions until the time of ICD removal (Hayse-Gregson 1973). Resolution of haemopneumothorax and removal of ICD was typically achieved after 2 days from insertion and patients were discharged home within 3.6 days (Hayse-Gregson 1973).

Another South African paper investigated the timing of initiation of physiotherapy services for patients with penetrating stab injuries to the chest after placement of ICD for management of haemopneumothorax (Senekal & Eales 1994). In this small randomised trial, patients were allocated to either a group that received physiotherapy immediately after stabilisation of vital signs and insertion of ICD (day or night) or to a group that received physiotherapy during normal working hours only (Senekal & Eales 1994). Physiotherapy intervention was standardised across both groups and included diaphragmatic breathing; localised breathing; active coughing; active exercises of the shoulder and trunk; and marching on the spot for 1 min which was progressed to brisk walking and finally, running up and down a flight of stairs for 2 min (Senekal & Eales 1994). Twice daily physiotherapy resulted in a significantly shorter duration to haemothorax resolution (40 vs. 66 h, *p* = 0.0001) and shorter hospital LOS (43.9 vs. 77.5 h, *p* = 0.0001) with fewer cases presenting with a spike in temperature (2 vs. 8, *p* = 0.02) for those who received physiotherapy immediately after ICD insertion. A similar study design and intervention was used by Ngubane, De Charmoy and Eales (1999).

In this cohort study, physiotherapy treatment was provided once daily to both groups (immediate physiotherapy group and delayed physiotherapy group). Results showed that those who received immediate physiotherapy following stab injury to the chest had a significantly shorter haemothorax drainage time and hospital LOS compared with those who received delayed initiation of physiotherapy service (2.35 vs. 7.55 days). In addition, total cost related to physiotherapy service provision and hospital stay was R78 728 higher for the delayed physiotherapy group (Ngubane et al. 1999).

The management of patients with blunt chest trauma through multidisciplinary clinical pathways or care bundles that include respiratory support, analgesia, complication prevention and surgical fixation is effective in reducing morbidity and mortality (Kourouche et al. 2018). Physiotherapy forms an important and integral part of such service provision (Curtis et al. 2016; Kourouche et al. 2018; Unsworth et al. 2015), but details of the specific physiotherapy interventions used to obtain optimal patient recovery are often lacking in the available literature. Components of physiotherapy management described as part of these care bundles include incentive spirometry and deep breathing exercises (Kourouche et al. 2018; Unsworth et al. 2015). Incentive spirometry is reported to be effective in identifying patients with blunt chest trauma who are at risk of deterioration of their respiratory function, and deep breathing exercises seem to be effective in lowering patients’ perception of pain in the presence of effective analgesic therapy (Kourouche et al. 2018; Unsworth et al. 2015).

A global survey on the physiotherapy management of patients with major chest trauma was recently published (Van Aswegen et al. 2019). This survey investigated the current interventions and outcome measures used by experienced physiotherapists in patient management and differences in physiotherapy practice between geographical locations around the world (Van Aswegen et al. 2019). Experienced physiotherapists who work in major trauma centres were targeted to complete the survey and a response rate of 51% was achieved. The most frequently used interventions (> 80%) to optimise patients’ respiratory system function after major chest trauma were active cycle of breathing technique, deep breathing exercises, active coughing, forced expiratory technique, mobilisation and body positioning (Van Aswegen et al. 2019). These interventions require active patient participation and no specialised equipment to perform. An interesting finding was that physiotherapists who worked as part of a specialised trauma team used breath stacking (*p* = 0.03) and mechanical insufflation exsufflation (*p* = 0.03) more often for patient management than those who did not work as part of a specialised trauma team (Van Aswegen et al. 2019). Physiotherapy interventions reported to be less commonly used (< 35%) were manual chest therapy techniques, manual hyperinflation, ventilator hyperinflation, intermittent positive pressure breathing, inspiratory muscle training and NIV (Van Aswegen et al. 2019). Differences in the use of these management strategies existed between countries (Van Aswegen et al. 2019).

Some evidence from clinical trials and surveys exists to support the use of interventions such as incentive spirometry, deep breathing exercises, oscillating positive expiratory pressure therapy and early postoperative mobilisation to reduce the incidence of postoperative pulmonary complications in patients who underwent elective upper abdominal surgery (Do Nascimento et al. 2014; Haines, Skinner & Berney 2013; Patman et al. 2017; Silva, Li & Rickard 2013). A small pilot study investigated the effect of an enhanced recovery after surgery programme on clinical outcomes of patients with penetrating abdominal trauma. Early mobilisation formed part of this programme. Preliminary findings suggest that the enhanced recovery after surgery programme resulted in decreased hospital LOS without any increase in postoperative complications (Moydien et al. 2016). No other studies have reported on the types of physiotherapy interventions used or the effectiveness of these interventions on clinical outcomes of patients who received emergency abdominal surgery because of blunt or penetrating trauma.

### Physiotherapy interventions used to optimise musculoskeletal function

Body function and structure impairments (ICF) that patients with trunk trauma present with clinically include impairments in active shoulder joint and trunk range of motion (ROM) (pain from rib fractures, presence of ICD tubing and distention of the abdomen); temporary weakness of the arm on the affected side of the thorax (because of compression of ICD tubing on the first thoracic nerve of which the superior part joins the brachial plexus or compression on the third to sixth intercostal nerves which affects serratus posterior muscle action) (Glenesk & Lopez 2019); protective side-flexed trunk posture towards the affected side of the thorax (pain from rib fractures, ICD or postoperative); protective flexed trunk posture (postoperative abdominal pain); and generalised muscle wasting and weakness and poor cardiorespiratory exercise endurance in those who suffer more severe injury (Van Aswegen, Morrow & Van Aswegen 2015). The principles of physiotherapy management include prevention of joint stiffness through maintaining or restoring passive and active joint ROM of the trunk and all limbs, restoring independence in functional activities, restoring muscle power in all limbs and improving cardiorespiratory exercise endurance (Van Aswegen, Morrow & Van Aswegen 2015).

As mentioned earlier, Hayse-Gregson (1973), Senekal and Eales (1994) and Ngubane et al. (1999) placed much focus on early active exercise therapy involving the upper limbs and trunk in the management of patients with penetrating trunk trauma. The exercises described included active ROM activities and cardiorespiratory exercise training such as marching on the spot, brisk walking, climbing and running up and downstairs. This approach to management resulted in shorter hospital LOS (Hayse-Gregson 1973; Ngubane, De Charmoy & Eales 1999; Senekal & Eales 1994).

Mobilisation activities such as sitting patients over the edge of the bed, sitting out of bed, sit to stand exercises and walking away from the bedside were used by the majority of participants in the global survey for the management of patients with major chest trauma (Van Aswegen et al. 2019). There were no significant differences among countries in the use of these mobilisation activities (Van Aswegen et al. 2019). Targeted exercises of the shoulder and trunk to gain active ROM and muscle power and high-level exercise training such as riding a stationary bicycle to address cardiorespiratory endurance were performed by fewer participants (Van Aswegen et al. 2019).

No evidence could be found on the use of exercise therapy and early mobilisation interventions in preventing or addressing musculoskeletal complications in patients who underwent emergency surgery because of blunt or penetrating injury to the abdomen. It is reasonable to assume that these patients have higher morbidity because of a larger extent of organ damage sustained from trauma and risk of sepsis and organ failure compared with those who undergo elective surgery. Therefore, the role of exercise interventions on recovery of musculoskeletal function (muscle power and endurance) in this patient population needs further examination.

### Physiotherapy interventions used to manage pain

The detrimental effects of pain on respiratory function in patients with trunk trauma have been described earlier in this review. In the clinical setting, physiotherapists are responsible for managing their patients’ pain through the use of educational strategies for self-empowerment and non-pharmacological interventions.

Education strategies used for patient management should include information on pain neuroscience and pain management methods such as deep breathing, relaxation and support applied to the wound or injured area during coughing and sneezing (Van Aswegen et al. 2019). Results from the global survey showed that units with a dedicated trauma physiotherapist used education strategies more frequently (*p* = 0.02) than units without a dedicated trauma physiotherapist (Van Aswegen et al. 2019).

Grammatopoulou et al. (2010) investigated the effect of the active cycle of breathing technique (ACBT), added to standard physiotherapy management (body positioning, incentive spirometry, supported coughing and early mobilisation) and analgesia administration on pain levels in adult patients with three or more rib fractures.

Active cycle of breathing technique was performed twice daily for the first 3 days after injury and then once daily from days 4 to 7. The authors showed a significant reduction in the level of pain experienced reported by the ACBT group compared with the standard therapy group from days 3 to 7 of hospitalisation. They postulated that the reduction in pain after ACBT could be ascribed to the anti-inflammatory and healing effects of exercise or to the gate control theory of pain and descending pain mechanisms (Grammatopoulou et al. 2010).

As mentioned earlier, deep breathing has been associated with a reduction in level of pain experienced by patients with major chest trauma in the presence of optimal analgesic management as part of management through multidisciplinary care bundles (Kourouche et al. 2018; Unsworth et al. 2015). Another non-pharmacological intervention that has been described for pain management in patients with rib fractures is transcutaneous electrical nerve stimulation (TENS) (Oncel et al. 2002). This randomised trial tested the effectiveness of TENS therapy compared with non-steroidal anti-inflammatory therapy (NSAIDS) or placebo in patients with uncomplicated minor rib fractures. The authors reported that TENS was more effective than NSAIDS or placebo in pain reduction on days 1 and 3 after injury (Oncel et al. 2002). Translation of these findings into clinical practice may be limited as patients with more severe chest trauma are usually encountered in acute care settings, and therefore, the effectiveness of TENS as a pain management strategy in these patients is unknown. Few experienced trauma physiotherapists (11%) reported using TENS frequently in their management of patients with major chest trauma (Van Aswegen et al. 2019).

The use of kinaesiotaping to support the chest wall in the presence of rib fractures has gained popularity in clinical settings over the years. Only two studies of low methodological quality and small sample sizes investigated the effect of kinaesiotaping on patients with undisplaced rib fractures (Czyzewski et al. 2012; Sareen, Jain & Pagare 2015). Few experienced trauma physiotherapists (6%) reported using kinaesiotaping frequently as part of patient management following major chest trauma (Van Aswegen et al. 2019). Thus, the effectiveness of kinaesiotaping as a pain management strategy in this patient population cannot be confirmed.

### Quality of life of survivors of trunk trauma and rehabilitation service provision

As patient management strategies evolve over time, the majority of those who sustain trunk trauma survive beyond hospital discharge (Baker et al. 2018). It, therefore, becomes important to understand the impact that their body function and structure impairments and activity limitations, experienced during their hospital stay, may have on their level of participation and reintegration into society and their QOL.

A cohort study on the characteristics of chest wall injuries reported that at 2 months after discharge, patients with fractures of the first four ribs present with significant reductions in QOL related to physical function (Dhillon et al. 2015). Those with fractures of ribs 9–12 present with significantly worse respiratory symptoms such as shortness of breath and reductions in QOL related to physical function (Dhillon et al. 2015).

Patients who had prolonged mechanical ventilation (> 5 days) and penetrating trunk trauma present with significant limitations in exercise capacity, upper and lower extremity muscle strength and QOL related to physical function up to 6 months after hospital discharge compared with those who had penetrating trunk trauma but a shorter duration of mechanical ventilation (Van Aswegen et al. 2010; Van Aswegen et al. 2011).

There was a moderate negative association between muscle strength in the long ventilation group and their ICU and hospital LOS. Higher scores for morbidity were associated with decreased distances walked by this group on the 6-min walk test at 3 and 6 months after discharge (Van Aswegen et al. 2010). A cross-sectional study on the QOL of trauma survivors showed that those with blunt or penetrating trauma presented with some problems related to performing usual activities, pain and discomfort and anxiety and depression at 6 months after discharge (Schneiderman, Van Aswegen & Becker 2013).

A longitudinal study investigated physical function and pain after surgical or conservative management of patients with multiple rib fractures over a 12-month period (Fagevik Olsén et al. 2016). Those who received conservative management presented with significantly reduced thoracic excursion and thoracic ROM, more kinaesiophobia and more discomfort in ‘sitting’ and ‘standing bent over a sink’ at 1 year following discharge than those who received surgical fixation of the chest wall (Fagevik Olsén et al. 2016). However, others were unable to demonstrate any QOL benefits of surgical rib fixation over conservative management for patients with multiple rib fractures when assessed 2 years after hospital discharge (Marasco et al. 2019).

A recent narrative literature review on long-term outcomes and QOL of patients following blunt chest trauma reported that unresolved ongoing pain, reduced physical and respiratory function and chest wall deformity persisted at 24 months after discharge (Baker et al. 2018). Key predictors of negative long-term patient outcomes identified by the authors were severity of injury and presence of a flail chest (Baker et al. 2018).

Baker et al.. 2018 stated that:

The evidence included in this review highlights the need for clinicians to consider the long-term outcomes of trauma patients when planning care and assessing ongoing care needs beyond the acute hospital admission. (p. 14)

A qualitative study explored the perceptions of patients with multiple rib fractures on their journey to recovery (Claydon et al. 2018). Participants reported that they struggled with breathing and pain, felt that their lives were on hold and that they felt lucky to be alive. The authors emphasised that rehabilitation and patient education after rib fracture injury should focus on pain management, cardiorespiratory fitness and emotional well-being (Claydon et al. 2018).

No information could be found on the presentation of and QOL of patients recovering from blunt or penetrating trauma to the abdomen.

From the above information, it is apparent that survivors of trunk trauma, and particularly chest trauma, struggle with limitations in physical function up to 2 years following discharge. Results from the global survey showed that only 8% of participants provided post-discharge rehabilitation services to patients following major chest trauma in the form of functional and strengthening exercises (Van Aswegen et al. 2019).

No clinical trials were identified that investigated post-discharge rehabilitation service provision to this patient population.

### Outcome measures

In clinical practice, it is important to assess a patient’s response to treatment received and to determine whether progression of treatment is appropriate or not. Outcome measures are used for such purposes. Table 2 displays the outcome measures that have been used in clinical trials in the blunt and penetrating trunk trauma population (Baker et al. 2018; Czyzewski et al. 2012; Dhillon et al. 2015; Fagevik Olsén et al. 2016; Grammatopoulou et al. 2010; Sareen et al. 2015; Schneiderman et al. 2013; Van Aswegen et al. 2010, 2011; Whelan, Van Aswegen & Corner 2018).

**TABLE 2:** Outcome measures used in trunk trauma research.

Impairment	Outcome measure	Source (Reference)
Dyspnoea	Modified Medical Research Council Dyspnoea Scale	Dhillon et al. 2015
Exercise endurance	6-min walk test	Van Aswegen et al. 2010
Kinesiophobia	Tampa score	Fagevik Olsén et al. 2016
Lung function	Forced expiratory volume in 1 s (FEV_1_)	Fagevik Olsén et al. 2016; Van Aswegen et al. 2010
	Forced vital capacity (FVC)	
	FEV_1_/FVC	
	Residual volume	
	Total lung capacity	
	Peak expiratory flow	
Muscle strength	Hand held dynamometer	Van Aswegen et al. 2010
Pain	Visual analogue scale	Baker et al. 2018; Czyzewski et al. 2012; Grammatopoulou et al. 2010; Sareen et al. 2015
	McGill Pain questionnaire	
	Numeric Pain Rating Scale	
Physical function	Chelsea Critical Care Physical Assessment (CPAx) tool	Baker et al. 2018; Fagevik Olsén et al. 2016; Whelan et al. 2018
	Disability Rating Index	
	Karnofsky Performance Scale	
Quality of life (physical function and mental health)	EQ-5D	Baker et al. 2018; Dhillon et al. 2015; Schneiderman, Van Aswegen & Becker 2013; Van Aswegen et al. 2011
	Short Form-36 Health Survey	
	Short Form-12 Health Survey	
	St George’s Respiratory questionnaire	
Shoulder ROM	Goniometry	Fagevik Olsén et al. 2016
Shoulder function	Bostrӧm index	Fagevik Olsén et al. 2016
Thoracic expansion and thoracic ROM	Tape measure	Fagevik Olsén et al. 2016

ROM, range of motion.

Few experienced trauma physiotherapists reported using outcome measures as part of their daily clinical practice (Van Aswegen et al. 2019). Those who did use outcome measures mostly used the Chelsea Critical Care Physical Assessment (CPAx) tool for the assessment of physical function and the 6-min walk test for assessment of exercise capacity (Van Aswegen et al. 2019).

It seems that there is a lack of use of outcome measures in clinical practice when compared with its use for research purposes. The evidence shared in this review shows that patients who are recovering from thoracic trauma or trunk trauma suffer with chronic pain which has a significant influence on their long-term QOL. Few instances are post-discharge rehabilitation services provided to these survivors. If outcome measures are not utilised by physiotherapists in clinical practice to identify a particular patient’s needs in relation to management of chronic pain and reintegration back into the community and the work force of a country, provision of post-discharge rehabilitation services cannot be motivated for and these patients’ QOL will remain suboptimal. Reasons for the apparent lack of use of outcome measures as a part of daily clinical practice should be investigated to determine which modifiable factors influence their use by clinicians.

## Conclusions and recommendations

Physiotherapy management of patients with blunt or penetrating trunk trauma during hospitalisation and in the long term after discharge is a field of clinical practice that is rich for further research. The small numbers of available studies of low-to-moderate quality support the use of interventions such as mobilisation away from the bedside, functional activities in and out of bed, deep breathing exercises, active coughing and physiotherapy as part of multidisciplinary care pathways for those with major chest trauma to reduce their morbidity and shorten their hospital LOS. Little is currently known regarding physiotherapy management of patients with traumatic abdominal injury. Survivors of trunk trauma do not make a complete recovery and suffer limitations in physical-function-related QOL long after hospital discharge.

Further research in this field should focus on investigating the factors that affect physiotherapy service provision during and after hospital discharge and its cost implications; the effects that specific interventions have on patient outcomes and LOS (e.g. targeted shoulder and trunk exercises; high-impact exercise training such as cycling and running up a flight of stairs); and investigating the clinical outcomes of patients recovering from blunt or penetrating abdominal trauma and the impact that physiotherapy has on influencing these outcomes to optimise patient recovery.
